# Estimating mutual information using B-spline functions – an improved similarity measure for analysing gene expression data

**DOI:** 10.1186/1471-2105-5-118

**Published:** 2004-08-31

**Authors:** Carsten O Daub, Ralf Steuer, Joachim Selbig, Sebastian Kloska

**Affiliations:** 1 Max Planck Institute of Molecular Plant Physiology, Potsdam, 14424, Germany; 2 Nonlinear Dynamics Group, Institute of Physics, University of Potsdam, Potsdam, 14415, Germany; 3 Scienion AG, Volmerstrasse 7a, Berlin, 12489, Germany; 4 Center for Genomics and Bioinformatics, Karolinska Institutet, Stockholm, 17177, Sweden

## Abstract

**Background:**

The information theoretic concept of mutual information provides a general framework to evaluate dependencies between variables. In the context of the clustering of genes with similar patterns of expression it has been suggested as a general quantity of similarity to extend commonly used linear measures. Since mutual information is defined in terms of discrete variables, its application to continuous data requires the use of binning procedures, which can lead to significant numerical errors for datasets of small or moderate size.

**Results:**

In this work, we propose a method for the numerical estimation of mutual information from continuous data. We investigate the characteristic properties arising from the application of our algorithm and show that our approach outperforms commonly used algorithms: The significance, as a measure of the power of distinction from random correlation, is significantly increased. This concept is subsequently illustrated on two large-scale gene expression datasets and the results are compared to those obtained using other similarity measures.

A C++ source code of our algorithm is available for non-commercial use from kloska@scienion.de upon request.

**Conclusion:**

The utilisation of mutual information as similarity measure enables the detection of non-linear correlations in gene expression datasets. Frequently applied linear correlation measures, which are often used on an ad-hoc basis without further justification, are thereby extended.

## Background

The evaluation of the complex regulatory networks underlying molecular processes poses a major challenge to current research. With modern experimental methods in the field of gene expression, it is possible to monitor mRNA abundance for whole genomes [1,2]. To elucidate the functional relationships inherent in this data, a commonly used approach is the clustering of co-expressed genes [3]. In this context, the choice of the similarity measure used for clustering, as well as the clustering method itself, is crucial for the results obtained. Often, linear similarity measures such as the Euclidean distance or Pearson correlation are used in an ad-hoc manner. By doing so, it is possible that subsets of non-linear correlations contained in a given dataset are missed.

**Figure 1:**
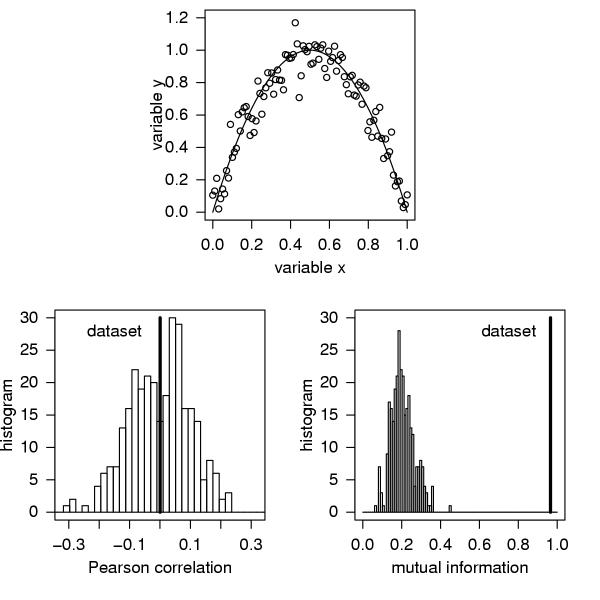
Two datasets *X *and *Y *(100 data points) show a hypothetical dependency *f*(*x*) = 4*x*(1 - *x*) (top). The Pearson correlation coefficient is not able to detect a significant correlation as shown in the histogram plot of the dataset compared to 300 realisations of shuffled data (left). Mutual information clearly shows that the two datasets are not statistically independent (right).

Therefore, information theoretic concepts, such as mutual information, are being used to extend more conventional methods in various contexts ranging from expression [4-8] and DNA sequence analysis [9,10], to reverse engineering [11] and independent component analysis [12,13]. Also aside the bioinformatics field, mutual information is widely utilised in diverse disciplines, such as physics [14], image recognition [15], speech recognition [16], and various others. In extension to other similarity measures, mutual information provides a general measure of statistical dependence between variables. It is thereby able to detect any type of functional relationship, extending the potentialities of linear measures as illustrated in Figure 1.

In this work, we discuss mutual information as a measure of similarity between variables. In the first section, we give a short introduction into the basic concepts including a brief description of the commonly used approaches for numerical estimation from continuous data. In the following section, we then present an algorithm for estimating mutual information from finite data.

The properties arising from this approach are compared to previously existing algorithms. In subsequent sections, we then apply our concept to large-scale cDNA abundance datasets and determine if these datasets can be sufficiently described using linear measurements or if a significant amount of non-linear correlations are missed.

### Mutual information

Mutual information represents a general information theoretic approach to determine the statistical dependence between variables. The concept was initially developed for discrete data. For a system, *A*, with a finite set of *M *possible states {*a*_1_, *a*_2_, ... , 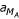}, the Shannon entropy *H*(*A*) is defined as [17]


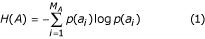


where *p*(*a_i_*) denotes the probability of the state *a_i_*. The Shannon entropy is a measure for how evenly the states of *A *are distributed. The entropy of system *A *becomes zero if the outcome of a measurement of *A *is completely determined to be *a_j_*, thus if *p*(*a_j_*) = 1 and *p*(*a_i_*) = 0 for all *i *≠ *j*, whereas the entropy becomes maximal if all probabilities are equal. The joint entropy *H*(*A, B*) of two systems *A *and *B *is defined analogously


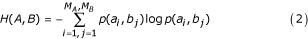


This leads to the relation

*H*(*A, B*) ≤ *H*(*A*) + *H*(*B*)     (3)

which fulfils equality only in the case of statistical independence of *A *and *B*. Mutual information *MI*(*A, B*) can be defined as [17]

*MI*(*A, B*) = *H*(*A*) + *H*(*B*) - *H*(*A, B*) ≥ 0     (4)

It is zero if *A *and *B *are statistically independent and increases the less statistically independent *A *and *B *are.

If mutual information is indeed to be used for the analysis of gene-expression data, the continuous experimental data need to be partitioned into discrete intervals, or bins. In the following section, we briefly review the established procedures; a description of how we have extended the basic approach will be provided in the subsequent section.

### Estimates from continuous data

In the case of discrete data the estimation of the probabilities *p*(*a_i_*) is straightforward. Many practical applications, however, supply continuous data for which the probability distributions are unknown and have to be estimated. In a widely used approach [7], the calculation of mutual information is based on the binning of data into *M *discrete intervals *a_i_*, *i *= 1... *M_A_*. For experimental data consisting of *N *measurements of a variable *x_u_*, *u *= 1... *N*, an indicator function Θ_*i *_counts the number of data points within each bin. The probabilities are then estimated based on the relative frequencies of occurrence


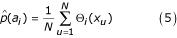


with


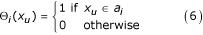


For two variables the joint probabilities 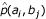 are calculated analogously from a multivariate histogram. Additionally it has been suggested [14] to adaptively choose the sizes of the bins, so that each bin constructed nearly has a uniform distribution of points. In a different approach, kernel methods are used for the estimation of the probability density of Eq. (5) [18-20]. Entropies are then calculated by integration of the estimated densities. Recently, an entropy estimator 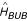 was suggested [21] and showed in an extensive comparison to other commonly used estimators to be superior.

## Results

### Fuzzy mutual information

**Figure 2:**
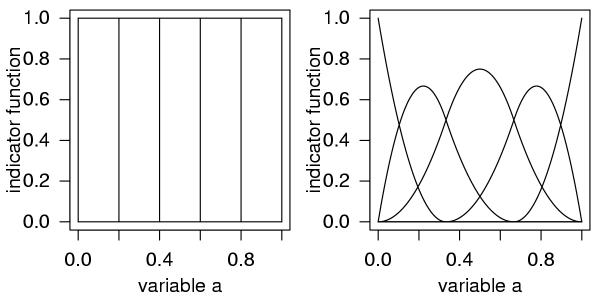
The continuous experimental data for the variable *x *needs to be binned for the calculation of mutual information. The indicator function of Eq. (5) counts the number of data points within each bin (example with *M*_*x *_= 5 bins, left). The generalised indicator function based on B-spline functions of Eq. (8) extends the bins to polynomial functions (example with *M*_*x *_= 5 bins and spline order *k *= 3, right). The bins now overlap and the weight of each data point to each of the bins is given by the value of the respective B-spline functions at the data point. By definition, all weights contributing to one data point sum up to unity.

In the classical binning approach, described above, each data point is assigned to one, and only one, bin. For data points near to the border of a bin, small fluctuations due to biological or measurement noise might shift these points to neighbouring bins. Especially for datasets of moderate size, the positions of the borders of the bins can thereby strongly affect the resulting mutual information [18]. In a manner analogous to kernel density estimators (KDE), we now present a generalisation to the classical binning in which we aim to overcome some of the drawbacks associated with the simple approach. Within our algorithm, we allow the data points to be assigned to several bins simultaneously. For this, we extended the indicator function Θ(*x*) to the set of polynomial B-spline functions. Here, we do not provide the mathematical details for these functions since they have been discussed extensively in the literature [22-24], but rather focus on the practical applicability. Within the B-spline approach, each measurement is assigned to more than one bin, *i*, with weights given by the B-spline functions *B*_*i,k*_. The spline order *k *determines the shape of the weight functions and thereby the number of bins each of the data points is assigned to. A spline order *k *= 1 corresponds to the simple binning, as described in the previous section: Each data point is assigned to exactly one bin (Figure 2, left). For *k *= 3, each data point is assigned to three bins, with the respective weights given by the values of the B-spline functions at the data point (Figure 2, right).

### B-spline functions

The first step in the definition of the B-spline functions is the definition of a knot vector *t*_*i *_for a number of bins *i *= 1... *M *and one given spline order *k *= 1... *M *- 1 [22]


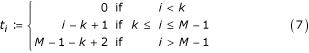


where the spline order determines the degree of the polynomial functions. The domain of the B-spline functions lies in the interval *z *∈ [0, *M *- *k *+ 1]. To cover the range of the variables, the new indicator function based on the B-spline functions needs to be linearly transformed to map their range. The recursive definition of the B-spline functions are as follows [22]


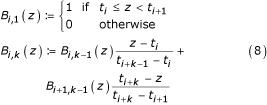


An important property of B-spline functions is the implicit standardisation of coefficients: All weights belonging to one data point sum up to unity.

### Algorithm

#### Input

• Variables *x *and *y *with values *x*_*u *_and *y*_*u*_, *u *= 1... *N*

• Bins *a*_*i*_, *i *= 1... *M*_*x *_and *b*_*j*_, *j *= 1... *M*_*y*_

• Spline order *k*

#### Output

• Mutual information between variable *x *and *y*

#### Algorithm

1. Calculation of marginal entropy for variable *x*

(a) Determine 
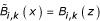
 with


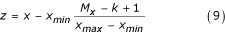


(b) Determine *M*_*x *_weighting coefficients for each *x*_*u *_from 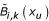


(c) Sum over all *x*_*u *_and determine *p*(*a*_*i*_) for each bin *a*_*i *_from


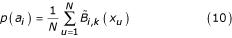


(d) Determine entropy *H*(*x*) according to Eq. (1)

2. Calculation of joint entropy of two variables *x *and *y*

(a) Apply steps 1 (a) and (b) to both variables *x *and *y*, independently

(b) Calculate joint probabilities *p*(*a*_*i*_, *b*_*j*_) for all *M*_*x *_× *M*_*y *_bins according to


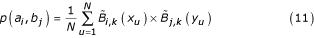


(c) Calculate the joint entropy *H*(*x,y*) according to Eq. (2)

3. Calculate the mutual information *MI*(*x,y*) according to Eq. (4)

### Example

We show the estimation with the standard binning and our approach ex-emplarily on two artificial variables *x *= 0.0,0.2,0.4,0.6,0.8,1.0 and *y *= 0.8,1.0,0.6,0.4,0.0,0.2 for *M *= 3 bins, spline order *k *= 2, and the logarithm to basis two.

#### Simple binning

For both variables, each of the three histogram bins contains two values *p*(*a*_1_) = *p*(*a*_2_) = *p*(*a*_3_) = 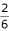, analogously for *p*(*b_i_*) due to the symmetry of data *H*(*x*) = *H*(*y*) = 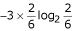 = log_2 _3 ≈ 1.58. For the calculation of the joint probability, three of the nine two dimensional bins contain two values each *p*(*a*_1_, *b*_3_) = *p*(*a*_2_, *b*_2_) = *p*(*a*_3_, *b*_1_) = 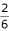 resulting in *H*(*x, y*) = log_2 _3 and *MI*(*x, y*) = log_2 _3.

#### B-spline approach

The modified indicator function 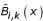 is determined to *B*_*i,k*_(2*x*) according to Eq. (9) (rule 1(a)). For each value *x*_*u *_three weighting coefficients are determined (rule 1(c)) and probabilities are calculated (rule 1(d)) (Table 1). The analogous procedure is applied to variable *y *and the single entropies are calculated to *H*(*x*) = *H*(*y*) = Iog_2_(10) - 0.61og_2_(3) - 0.41og_2_(4) ≈ 1.57. Both, *H*(*A*) and *H*(*B*), are slightly smaller than the entropies calculated from the simple binning. The joint probabilities are *p*(*a*_1_, *b*_1_) = *p*(*a*_3_, *b*_3_) = 0, *p*(*a*_1_, *b*_2_) = *p*(*a*_2_, *b*_1_) = *p*(*a*_2_, *b*_3_) = *p*(*a*_3_, *b*_2_) = 0.56/6, *p*(*a*_1_, *b*_3_) = *p*(*a*_3_, *b*_1_) = 1.24/6, *p*(*a*_2_, *b*_2_) = 1.28/6 (rule 2 (b)) resulting in *H*(*x,y*) = 2.7 and *MI*(*x,y*) = 0.45.

In the next sections, we discuss some of the properties arising from the utilisation of B-spline functions for the estimation of mutual information and compare our approach to other commonly used estimators. We support this discussion using examples for which the underlying distributions and thereby the true mutual information is known.

### Size of data

It has been discussed elsewhere [25-28,20] that the estimated mutual information is systematically overestimated for a finite size of *N *data points. For the simple binning approach, the mean observed mutual information can be calculated explicitly as the deviation from the true mutual information


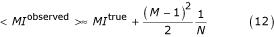


**Figure 3:**
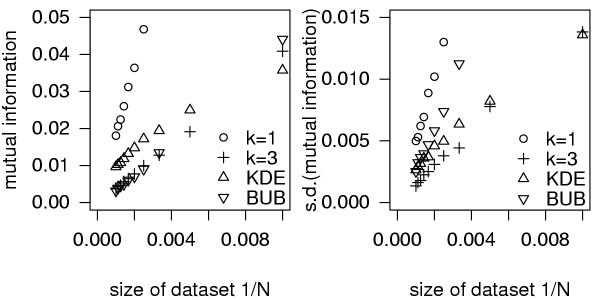
Mutual information is estimated for artificially generated equidis-tributed random numbers from the simple binning (*k *= 1), the B-spline approach (*k *= 3), and the entropy estimator 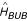 using *M *= 6 bins, and additionally from the kernel density estimator. The average over an ensemble of 600 trials is shown as a function of the size of the dataset (left) together with the standard deviation (right).

As can be seen for an example of artificially generated equidistributed random numbers (Figure 3, left), mutual information calculated from the simple binning scales linearly with 1/*N*, with the slope depending on the number of bins *M *in accordance with Eq. (12). Figure 3 shows that this scaling is preserved for the extension to B-spline functions, while the slope is significantly decreased for *k *= 3, compared to the estimation with the simple binning (*k *= 1). Mutual information calculated from KDE does not show a linear behaviour but rather an asymptotic one with a linear tail for large datasets. The values are slightly increased compared to the ones from the B-spline approach. The entropy estimator 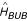 gives values comparable to the ones obtained from the B-spline approach.

More importantly, a similar result also holds for the standard deviation of mutual information. As shown in Figure 3 (right), the standard deviation of the mutual information estimated with the simple binning (*k *= 1) scales with 1/*N *for statistically independent events [26,29]. For the B-spline approach (*k *= 3), this scaling still holds, but the average values are decreased significantly. For the KDE approach, an asymptotic run above the values from the B-spline approach is observed, again with linear tail for large datasets. 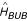 shows a linear scaling slightly below the simple binning.

### The spline order

The interpretation of any results obtained from the application of mutual information to experimental data is based on testing to see if the calculated results are consistent with a previously chosen null hypothesis. By following the intuitive approach that the null hypothesis assumes the statistical independence of variables, mutual information is tested against a surrogate dataset, which is consistent with this null hypothesis. As discussed previously in more detail [20], one way of generating such a surrogate dataset is by random permutations of the original data. From the mutual information of the original dataset *MI*(*X,Y*)^data^, the average value obtained from surrogate data <*MI*(*X*^surr^, *Y*^surr^) >, and its standard deviation σ^surr^, the significance *S *can be formulated as


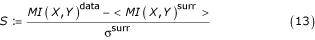


For each *S *the null hypothesis can be rejected to a certain level α depending on the underlying distribution. With increasing significance the probability of false positive associations drops.

**Figure 4:**
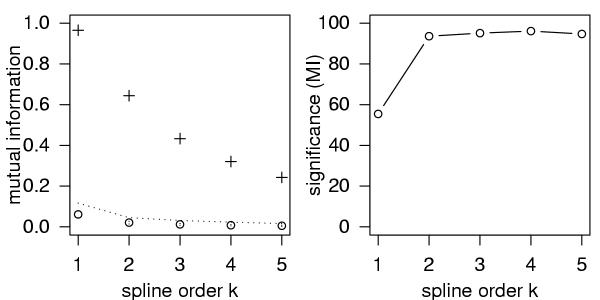
Mutual information calculated for a dataset of 300 data points drawn from the distribution shown in Figure 1 (crosses). The number of bins was fixed to *M *= 6. The average mutual information for 300 shuffled realisations of the dataset is shown (circles) together with the standard deviation as error-bars. The largest value found within the ensemble of shuffled data is drawn as a dotted line (left). The significance was calculated from Eq. (13) (right).

In the following, we address the influence of the spline order and the number of bins on the estimation of mutual information. Based on 300 data points of an artificially-generated dataset drawn from the distribution shown in Figure 1, we calculate the mutual information for *M *= 6 bins and different spline orders *k *= 1... 5 (Figure 4, left).

From 300 shuffled realisations of this dataset, the mean and maximum mutual information are shown with the standard deviation as error-bars. For all spline orders the null hypothesis can be rejected, in accordance with the dataset shown in Figure 1. To estimate the strength of the rejection, we calculate the significance according to Eq. (13) (Figure 4, right). It can be observed that the largest change in the significance of the mutual information occurs in the transition from *k *= 1 (simple boxes) to *k *= 2 with an increase by roughly two-fold. Using more sophisticated functions (*k *≥ 3) does not further improve the significance. Similar findings have been reported in the context of kernel density estimators [19]. The major contribution leading to this increase of the significance is given by the distribution of surrogate data which becomes more narrow for *k *> 1 leading to smaller standard deviations σ^surr^.

**Figure 5:**
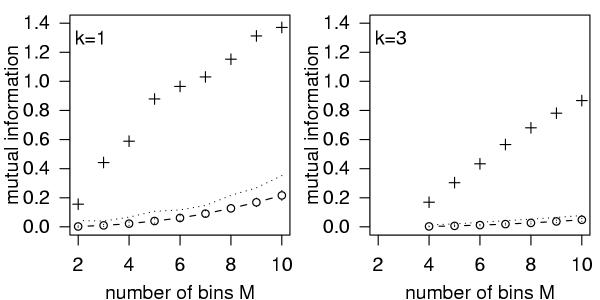
Based on the distribution of Figure 1, the mutual information for 300 data points and two spline orders *k *= 1 and *k *= 3 is shown as a function of the number of bins *M *(crosses) together with mean (circles) and standard deviations (error-bars) of 300 surrogates. The dotted lines indicate the largest mutual information found within the ensemble of surrogate data.

The same dataset is used to show the dependency of mutual information on the number of bins for two spline orders *k *= 1 and *k *= 3 (Figure 5). Mutual information estimated from data as well as from surrogate data shows a robust run without strong fluctuations within the range of bins shown. From this we can conclude that the choice of the number of bins does not affect the resulting mutual information notably as long as it is chosen to be within a reasonable range.

**Figure 6:**
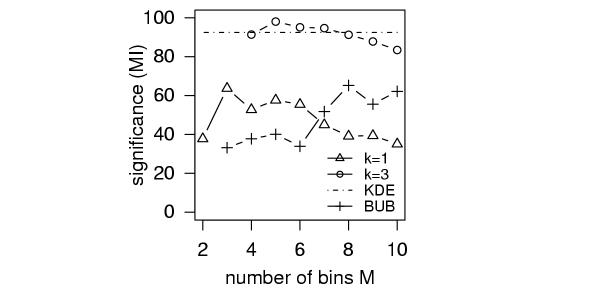
The significance, *S*, as a function of the number of bins, *M*, for the two examples of Figure 5, and for the entropy estimator 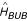. For kernel density estimators (KDE), the significance, which is not depending on *M*, is calculated to *S *= 92.

Again, the significance is calculated (Figure 6) and compared to the significances obtained from the KDE approach and the 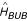 estimator. It can be observed that the significance of the mutual information calculated with B-spline functions increased roughly by two-fold compared to the simple binning. The significance obtained from KDE is not depending on *M *and was determined to be similar to the significance estimated from the B-spline approach. The numerically expensive integration of KDE, however, limits the size of utilisable datasets. The KDE run time requirements were 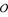
(10^4^) times higher than the ones from the B-spline approach. Strategies to simplify the integration step were proposed [20] but have to be used with caution since they assume particular properties of the distribution of experimental data that are in general not fulfilled. The recently introduced entropy estimator 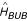 produces intermediate significances between the ones from the binning and the B-spline approach for higher bin numbers. For low bin numbers, the significances are relatively poor.

### Application on data

We now turn to the analysis of experimentally measured gene expression data. As shown previously, the application of mutual information to large-scale expression data reveals biologically-relevant clusters of genes [7,30]. In this section, we will not repeat these analyses, but determine if the correlations detected using mutual information are missed using the established linear measures.

**Figure 7:**
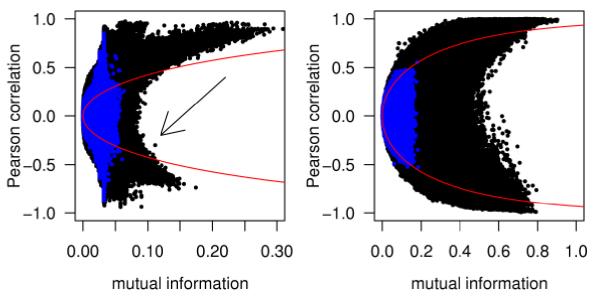
The Pearson correlation coefficient and the mutual information for all pairwise comparisons of genes for two large-scale gene expression datasets are shown (black points) overlayed by the same measures obtained from shuffled data (blue points). The expected mutual information calculated from Eq. (14) is shown as read curve. For the first dataset (left) genes containing undefined values were omitted resulting in 5345 genes measured under 300 experimental conditions [31]. For the second dataset (right) containing 22608 genes measured under 102 experimental conditions [33], a representative fraction is shown.

Among the most frequently used measures of similarity for clustering co-expressed genes are the Euclidean distance and the Pearson correlation coefficient *R *[3]. If correlations are well described by the Pearson correlation and the distribution of data is approximately Gaussian like, the relationship between the mutual information and the Pearson correlation given by [32]


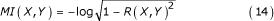


is expected to be fulfilled. Therefore, we calculated both, the mutual information and the Pearson correlation, for two large-scale gene expression datasets (Figure 7). For each pair of genes *X *and *Y *we plot the tuple (*MI*(*X,Y*), *R*(*X,Y*)). In order to address significance, we additionally calculate all tuples from shuffled data.

**Figure 8:**
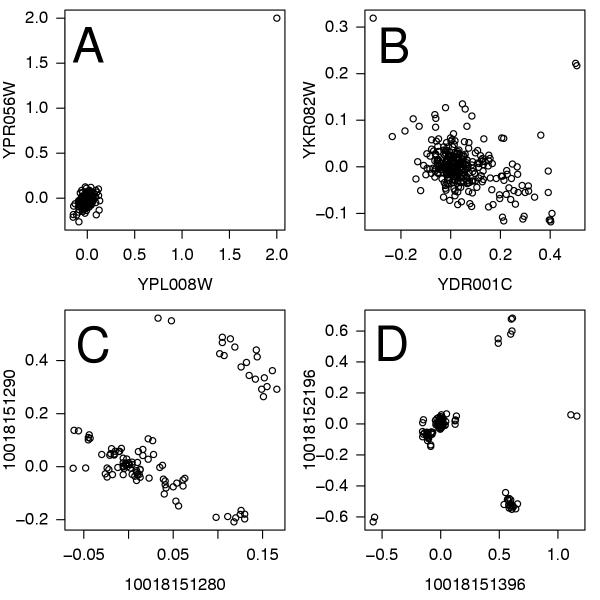
Examples of gene-gene plots for genes *X *and *Y *are shown for characteristic tuples (*MI*(*X,Y*), *R*(*X,Y*)) detected in Figure (7). For the first gene expression dataset under consideration [31], no non-linear correlations are detected. Moreover, tuples with high Pearson correlation and low mutual information, examples A and B, resulting from outlying values are detected. For the second dataset [33], however, tuples with low Pearson correlation and high mutual information are observed, see examples C and D. Such non-linear correlations are missed by solely using linear correlation measures.

The first dataset contains cDNA measurements for *S. cerevisiae *for up to *E*_1 _= 300 experiments [31]. To avoid numerical effects arising from different numbers of defined expression values (missing data points) for each gene, we exclusively utilised genes that are fully defined for all experimental conditions resulting in *G*_1 _= 5345 genes. Analysis on this dataset using mutual information has been done before [20,32] on rank-ordered data. The rank-ordering lead to homogeneously distributed data and thereby enabled the application of a simplified algorithm for the numerical estimation from kernel density estimators. The utilisation of our B-spline approach allows us to extend this analysis to non rank-ordered data thereby keeping the original distribution of experimental data. In contrast to the previous studies we find for non rank-ordered data that the theoretical prediction of Eq. 14 is no longer a lower bound for the comparison. Many tuples with high Pearson correlation but low mutual information can be detected arising from outlying expression values (Figure 8A). However, pairs of genes with high mutual information and low Pearson correlation, thus indicating a non-linear correlation, are not observed. The only remarkable tuple (marked with an arrow in Figure 7 and shown in Figure 8B) also arises from outlying values.

The second dataset contains cDNA measurements for *E*_2 _= 102 experiments on G_2 _= 22608 genes derived from 20 different human tissues [33]. In contrast to the first dataset, tuples with low Pearson correlation but high mutual information are indeed detected. For two exemplary chosen tuples (Figure 8C and 8D), clusters of experimental conditions can be clearly detected by eye. Such type of correlations are missed by analyses based exclusively on linear measures, such as the the analysis done in the original publication of this dataset.

**Figure 9:**
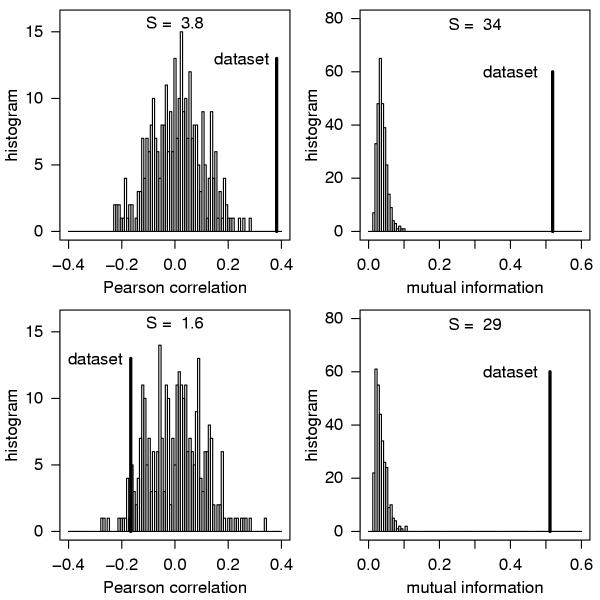
Significance values for the two gene-gene comparisons shown in Figure 8, C and D (top and bottom, respectively) are calculated from 300 shuffled realisations based on the Pearson correlation coefficient (left) and the mutual information (right) as distance measures.

For both datasets, tuples calculated from shuffled data (Figure 7, blue data points) result in small values for both similarity measures. Thereby, they indicate a high significance of the original associations. Peaks with high Pearson correlation in the first dataset arise from gene-gene associations with outlying values. Significance values for the exemplarily chosen pairs of genes of the second dataset (Figure 8C, and 8D) were explicitly calculated (Figure 9). They show high significance values for the two examples of observed non-linear correlations on the basis of the mutual information. Compared to this, the significances calculated from the Pearson correlation are poor. In summary, our analysis confirms for the first dataset that the Pearson correlation does not miss any non-linear correlations. As a side effect we are able to detect gene-gene pairs containing outlying values. For the second dataset, however, a substantial amount of non-linear correlations was detected. Gene-gene pairs exemplarily chosen from this fraction show a clustering of data points (experiments) with a high significance. Even though such patterns can be easily found by eye, computational methods need to be applied for the inspection of several hundred million comparisons.

## Discussion and conclusion

After a brief introduction into the information theoretic concept of mutual information, we proposed a method for its estimation from continuous data. Within our approach, we extend the bins of the classical algorithm to polynomial B-spline functions: Data points are no longer assigned to exactly one bin but to several bins simultaneously, with weights given by the B-spline functions. By definition, the weighting coefficients for each data point automatically sum up to unity. Though our algorithm is reminiscent of kernel density estimators [18], it keeps the basic idea to associate data points to discrete bins. In this way, we are able to avoid time-consuming numerical integration steps usually intrinsic to estimates of mutual information using kernel density estimators [20].

To show that our approach improves the simple binning method and to compare it to KDE and the recently reported estimator 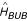, we provided a systematic comparison between all these algorithms for artificially generated datasets, drawn from a known distribution. We found that mutual information, as well as its standard deviation, scales linearly with the inverse size of a dataset for the standard binning method, for the B-spline approach, and for 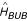. For the KDE approach we find an asymptotic behaviour with a linear tail for large datasets. Moreover, the discrimination of correlations from the hypothesis of statistical independence is significantly improved by extending the standard binning method to B-spline functions, as shown by a two-fold increase of the significance. Compared to KDE, the B-spline functions produce similar significances. However, due to the numerical expenses of the KDE, an application of this algorithm is limited to datasets of mod-erate size. The application of 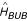 leads to significances in-between the standard binning and the B-spline approach for reasonable bin numbers. Linear correlation measures are among the most applied measures of similarity in the literature. Often, they are used on an ad-hoc basis and it is unclear whether a considerable number of non-linear correlations are missed. Here, we asked the question whether previous analyses, based on linear correlations, sufficiently described the correlations within gene expression datasets or whether mutual information detects additional correlations that are not detected by linear measures, such as the Pearson correlation. For data that is well described by the Pearson correlation, we can give the relation of the Pearson correlation to the mutual information explicitly [32]. Both measures were then applied to publicly available large-scale gene expression datasets [31,33]. We aimed to verify whether non-linear correlations shown as deviations from this relation can be detected.

Our findings show that the first dataset is fairly well described by the given relation of the Pearson correlation to the mutual information. No data points with high mutual information and low Pearson correlation are detected. Comparisons of genes containing outlying values, however, result in deviations with low mutual information and high Pearson correlation. From this, it follows that previous analyses on this dataset, based on Pearson correlation, did not miss any non-linear correlations. This presents an important finding since it is by all means supposable that the regulations inherent in the genetic network under consideration might show more complex behaviour than the observed linear ones. Even for one of the largest expression datasets at hand, insufficient data might complicate the detection of such complex patterns of regulation. Alternatively, the biological mechanisms which underlay the regulatory networks might not lead to non-linear correlations. It also has to be considered that the experimental methods applied for the generation of this dataset may make non-linear correlations difficult to detect. The second dataset, in contrast, reveals highly significant tuples with high mutual information and low Pearson correlation. Detailed gene-gene plots of such tuples show that the expression values of the contributing genes fall into groups of experimental conditions. Without attempting to draw conclusions about the biological context of such clusters here, they might reflect interesting situations worth to be analysed in detail.

## Authors' contributions

Most of the manuscript text was written by CD and edited by all authors. CD carried out the calculations and produced the figures. RS strongly contributed to the theoretical background of entropy and mutual information.

The implementation of the C++ program was carried out by SK. JS and SK supervised this work. All authors read and approved the final manuscript.

## Figures and Tables

**Table 1:** For the calculation of probabilities *p*(*a_i_*) according to the B-spline approach, *M*_*x *_weighting coefficients are determined for each value *x*_*u *_of variable *x*.

	*B*_*i*_=1,*k*=2(*x*_*u*_)	*B*_*i*_=2,*k*=2(*x*_*u*_)	*B*_*i*_=3,*k*=2(*x*_*u*_)
*x*_1_	1.0	0.0	0.0
*x*_2_	0.6	0.4	0.0
*x*_3_	0.2	0.8	0.0
*x*_4_	0.0	0.8	0.2
*x*_5_	0.0	0.4	0.6
*x*_6_	0.0	0.0	1.0
*p*(*a*_*i*_)	1.8/6	2.4/6	1.8/6
